# Automated selection of changepoints using empirical *P*-values and trimming

**DOI:** 10.1093/jamiaopen/ooac090

**Published:** 2022-10-29

**Authors:** Matthew Quinn, Arlene Chung, Kimberly Glass

**Affiliations:** Department of Biostatistics, Harvard T.H. Chan School of Public Health, Boston, Massachusetts, USA; Department of Biostatistics & Bioinformatics, Duke School of Medicine, Durham, North Carolina, USA; Department of Biostatistics, Harvard T.H. Chan School of Public Health, Boston, Massachusetts, USA; Channing Division of Network Medicine, Brigham and Women’s Hospital, Boston, Massachusetts, USA; Department of Medicine, Harvard Medical School, Boston, Massachusetts, USA

**Keywords:** mobile health, changepoints, time series, Monte Carlo method, regression

## Abstract

**Objectives:**

One challenge that arises when analyzing mobile health (mHealth) data is that updates to the proprietary algorithms that process these data can change apparent patterns. Since the timings of these updates are not publicized, an analytic approach is necessary to determine whether changes in mHealth data are due to lifestyle behaviors or algorithmic updates. Existing methods for identifying changepoints do not consider multiple types of changepoints, may require prespecifying the number of changepoints, and often involve nonintuitive parameters. We propose a novel approach, Automated Selection of Changepoints using Empirical *P*-values and Trimming (ASCEPT), to select an optimal set of changepoints in mHealth data.

**Materials and Methods:**

ASCEPT involves 2 stages: (1) identification of a statistically significant set of changepoints from sequential iterations of a changepoint detection algorithm; and (2) trimming changepoints within linear and seasonal trends. ASCEPT is available at https://github.com/matthewquinn1/changepointSelect.

**Results:**

We demonstrate ASCEPT’s utility using real-world mHealth data collected through the Precision VISSTA study. We also demonstrate that ASCEPT outperforms a comparable method, circular binary segmentation, and illustrate the impact when adjusting for changepoints in downstream analysis.

**Discussion:**

ASCEPT offers a practical approach for identifying changepoints in mHealth data that result from algorithmic updates. ASCEPT’s only required parameters are a significance level and goodness-of-fit threshold, offering a more intuitive option compared to other approaches.

**Conclusion:**

ASCEPT provides an intuitive and useful way to identify which changepoints in mHealth data are likely the result of updates to the underlying algorithms that process the data.

## INTRODUCTION

Recently, mobile health (mHealth) has taken on growing importance in medicine and public health, among other fields.^1–3^ mHealth devices, such as Fitbit smartwatches, often produce time series data by recording variables, like heart rate (HR) and number of steps, at regular intervals (eg, hourly or daily). Studying these data can bring important insights into how health changes over time. For instance, an individual might walk less after an injury. This type of event corresponds with a “changepoint,” a time at which the distribution of data changes, and typically reflects a change in the mean of the data, or a “mean-shift.” When attributable to lifestyle or behavior, these shifts often follow trends. For instance, an individual’s steps may follow a linear trend, increasing at a fixed rate as they train for a marathon. Alternatively, their steps may follow a seasonal trend, increasing and decreasing periodically as they walk more during the summer than the winter each year. However, in addition to lifestyle or behavioral changes, wearable devices also have both planned software or hardware updates as well as unexpected technical issues that can impact data collection and reporting. These can introduce relatively sudden “technological changepoints” to the data, which can be difficult to distinguish from behaviorally driven changes, obscuring patterns of interest. Therefore, it is necessary to identify and correct for these technological changepoints before proceeding with downstream analysis.

Unfortunately, mHealth device manufacturers often do not publicize the timing of planned updates and identified technological issues. Although an investigator could potentially monitor a manufacturer's release notes to determine when updates are pushed or manually inspect the mHealth data to find potential technological changepoints, these approaches are neither scalable nor practical and are especially challenging when studies utilize multiple types of devices. Even a single manufacturer may not push updates to all devices simultaneously, or they may require users to first update an associated smartphone app. Thus, manufacturer updates sometimes do not even coincide with a single timepoint across users.

There are many existing approaches that detect changepoints in time series by solving an optimization problem,^4^ including Pruned Exact Linear Time (PELT).^5^ Using PELT generally entails specifying an optimization penalty when detecting multiple changepoints, which is difficult to do in practice. Changepoints for a Range of PenaltieS (CROPS)^6^ allows one to efficiently run PELT under various penalties but does not select a final or optimal set of changepoints. Thus, instead of proposing another method for changepoint “detection,” we developed Automated Selection of Changepoints using Empirical *P*-values and Trimming (ASCEPT) to identify changepoints in mHealth data through changepoint “selection,” using relatively familiar statistical concepts instead of optimization parameters. ASCEPT analyzes multiple runs of PELT, considering iteratively larger sets of changepoints until the selected set no longer offers a statistically significant improvement over the prior set. Next, ASCEPT removes changepoints that are likely to be associated with lifestyle or behavioral changes rather than technological issues, ultimately yielding a single optimal set of changepoints. It is worth noting that ASCEPT shares similarities with circular binary segmentation (CBS),^7^ which, unlike many other changepoint detection algorithms, uses a statistical test to identify significant changepoints and then performs “pruning” to identify a subset of those changepoints. However, CBS was designed for genomics data and does not consider features common to mHealth data, such as seasonal patterns.

In addition to PELT and CBS, many other changepoint detection algorithms are available. For instance, there are changepoint algorithms that detect changes in linear, polynomial, and seasonal trends, rather than general mean-shifts,^8–10^ while other algorithms handle abrupt changes in the presence of trends, changes in variance, or changes in the presence of autocorrelated noise.^11–14^ While these algorithms address challenges similar to those found in mHealth data, ASCEPT addresses 2 problems that these algorithms do not. First, mHealth researchers typically have greater familiarity with statistical concepts than optimization concepts. ASCEPT replaces the choice of an optimization penalty, which is necessary for most changepoint detection algorithms, with a statistical metric. Second, mHealth time series can simultaneously contain sudden mean-shifts, linear trends, and seasonal trends, but existing algorithms tend to address only 1 or 2 of these characteristics at a time. Therefore, we designed ASCEPT to address all 3. ASCEPT also considers other details, such as how mHealth data may have as few as one observation between changepoints, which can prove difficult for many other algorithms. For a more detailed review of changepoint detection, please refer to the Supplementary Material.

In this study, we evaluate ASCEPT on both simulated data and Fitbit data collected by the Precision VISSTA study^15^ to determine whether the method appropriately identifies changepoints. Since ASCEPT and CBS^7^ share underlying principles for identifying changepoints but differ in their mechanics, we compare the performance of ASCEPT to that of CBS and examine whether ASCEPT provides better changepoint selection under comparable settings. Lastly, we perform a correction procedure to determine whether differences between the procedures have an impact when adjusting the mHealth data for the identified changepoints.

## MATERIALS AND METHODS

### Data

#### Precision VISSTA data

We evaluated the performance of ASCEPT on mHealth data from the Precision VISSTA study.^15^ This data set included adults in the United States who had inflammatory bowel diseases and were part of the parent Internet cohort study. Users participated in a survey and could donate their personal wearable device data towards research, meaning that the study followed a bring-your-own-device model. Thus, the data set contained multiple manufacturers and device types. Due to their prevalence in the cohort, we chose to focus on individuals who used a HR Fitbit device introduced in 2016–2019 (ie, the Alta HR, Blaze, Charge 2, Charge 3, Inspire HR, Ionic, Versa, or Versa 2), multiple Fitbit devices over time, or an unknown Fitbit device (eg, a Fitbit app). This subset of the data included 203 351 observations on 298 individuals recorded between May 15, 2015 and October 27, 2019.

These data included 6 activity variables (steps, distance, floors, elevation, calories, and time active) and 6 sleep variables (total sleep, deep sleep, light sleep, rapid eye movement (REM) sleep, time awake at night, and times woken). The median number of users contributing data on a given day ranged from 50 for REM, to 93–95 for the other sleep variables, to 131 for the activity variables. We excluded floors and elevation as their values largely stayed within narrow ranges near zero over the study period. We also excluded REM sleep due to a lack of any data between May 20, 2016 and March 26, 2017. For more information on the Precision VISSTA data, including preprocessing steps, see the Supplementary Materials.

**Figure 1.**
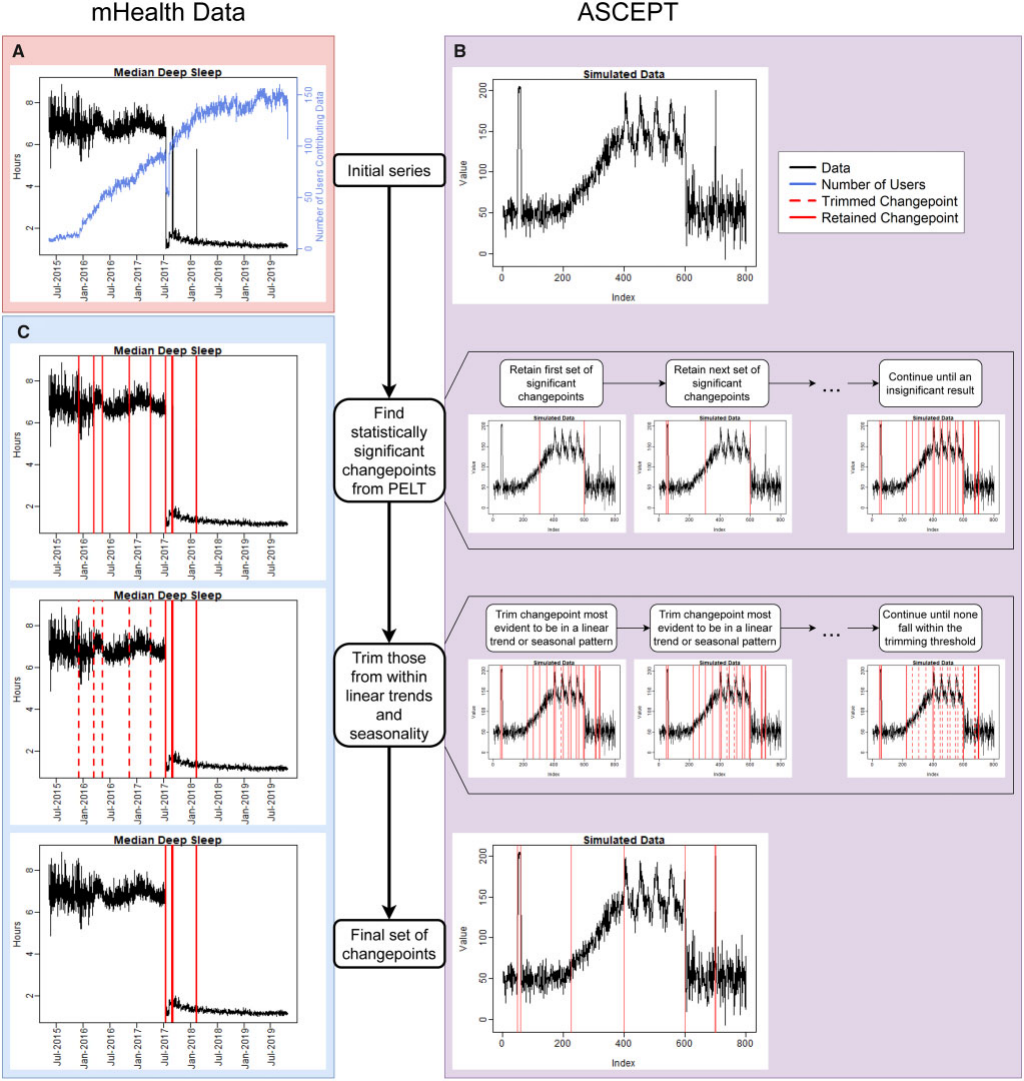
The ASCEPT workflow. (A) The daily median deep sleep from the Precision VISSTA study as well as the number of individuals contributing to these values. (B) ASCEPT broken down by stage and applied to simulated data. The first row shows the original simulated time series. The second row shows significant changepoints being iteratively identified. The third row shows changepoints within linear and seasonal trends being iteratively trimmed. The fourth row shows the simulated time series with the final set of identified changepoints. (C) The same results as (B) but for the deep sleep data.

To help identify population-level changepoints, we focused on the daily median value of each variable across users. As an example, Figure 1A shows the daily median amount of deep sleep across time, as well as the number of individuals contributing to these values. We observe that the median amount of deep sleep experienced an abrupt shift around July 2017. While it is possible that a single individual could have suddenly experienced large changes in deep sleep due to various life events, like an injury or the birth of a child, it is unlikely that the median deep sleep across many users truly decreased by 5–6 hours after July 19, 2017, only for it to later rebound multiple times. Instead, these shifts were more likely attributable to changes in how Fitbit’s algorithms calculated deep sleep. Thus, it is critical to identify and control for these technological changepoints in order to correctly describe human behavioral changes relevant to health and disease.

#### Simulated data

Shifts and patterns that appeared in the real data were often not defined well enough to serve as gold standards. For example, there appeared to be seasonality in the deep sleep data prior to July 19, 2017, but it was inconsistent (Figure 1A). Likewise, it was challenging to determine whether some points between May 15, 2015 and May 15, 2016 constituted behaviorally driven or technological changepoints because only 7–54 unique users contributed data during this time. On the order of tens of samples are generally necessary for a good approximation of a median value. Therefore, large behaviorally driven fluctuations were more likely during this earlier period compared to later, when up to 160 unique users contributed sleep observations (Supplementary Figure S1).

Due to these limitations, we first evaluated ASCEPT using a simulated time series containing 800 observations (Figure 1B). This data set had sudden mean-shift changepoints at indices 49, 60, 600, 699, and 700, an increasing linear trend between indices 201 and 400 inclusive, and a seasonal pattern between indices 401 and 600 inclusive.

### ASCEPT stage 1: Changepoint selection using empirical *P*-values

The first stage of ASCEPT incrementally accumulates mean-shift changepoints detected by PELT^5^ until the newly proposed changepoints do not offer a statistically significant improvement in goodness of fit.

**Figure 2.**
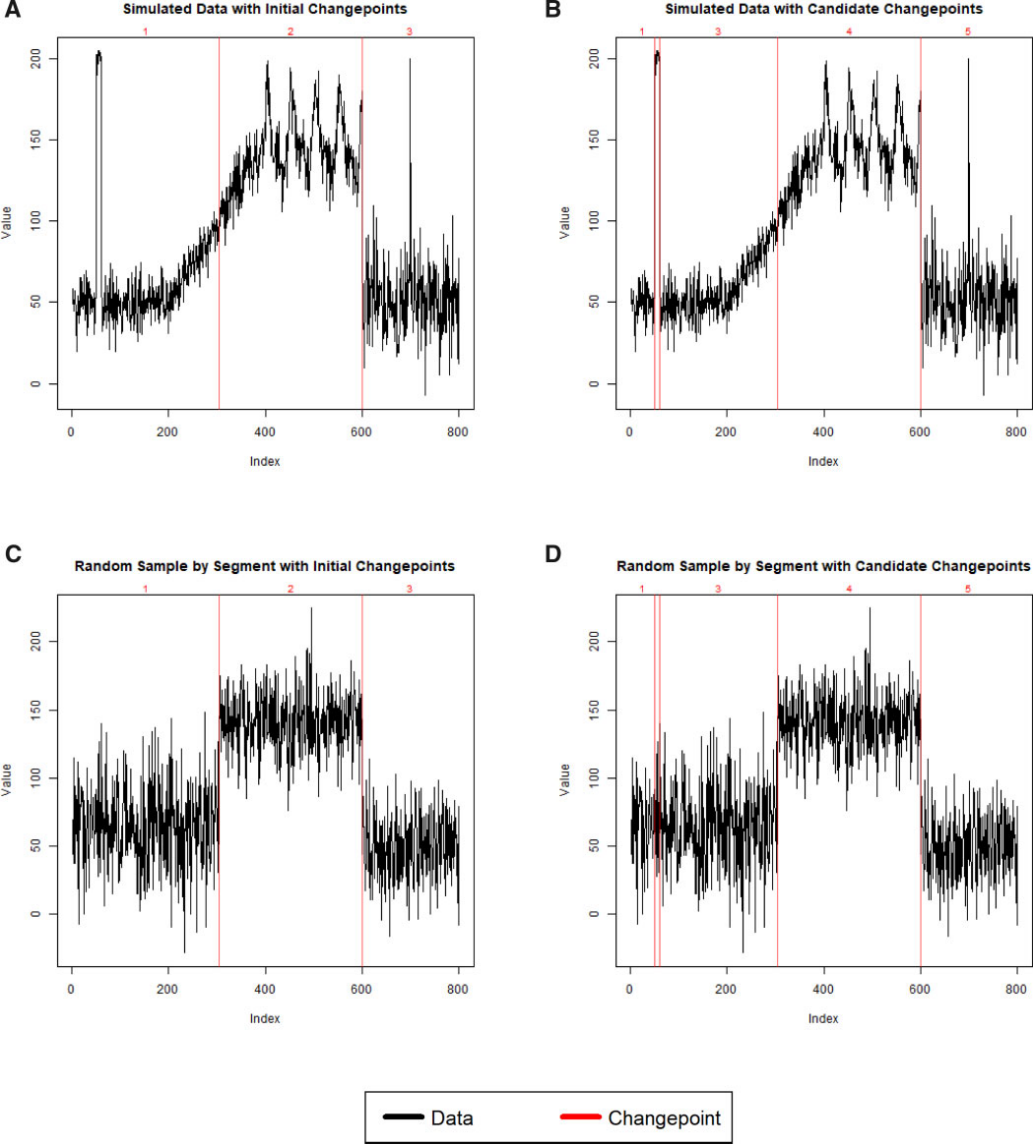
The process for assessing the significance of new changepoints in ASCEPT. (A) The simulated data with initial changepoints $T_{k}=\{305,\;600\}$. The log-likelihood, assuming independent and identically distributed observations within-segment, is −3727.3. (B) The simulated data set with the next set of changepoints $T_{k+1}=\{49,\;60,\;305,\;600\}$. The log-likelihood is −3512.3, thus the observed change in the log-likelihood is 215.0. (C) A Monte Carlo sample with the initial changepoints at {305, 600} shown. The observations in each segment are randomly drawn from a normal with a mean and standard deviation equal to that for the corresponding segment in (A). The log-likelihood is −3746.6. (D) The same Monte Carlo sample from (C) but now with the next set of changepoints at {49, 60, 305, 600} shown. The log-likelihood is −3745.0. The change in the log-likelihood for this Monte Carlo sample under the null is therefore 1.6. The process in (C) and (D) is repeated a large number of times to generate an empirical null distribution for the change in the log-likelihood. In all plots, the segments between the identified changepoints are numbered.

We use CROPS^6^ to efficiently run PELT for a range of different optimization penalties. From this, we obtain various sets of changepoints, from those found under high penalties to those found under low penalties. We let a changepoint at position j indicate that the time series' distribution changes between j and j+1. Let $T_{k}$ denote the kth set of changepoints, k=0, 1, …, K, and let $T_{0}=\emptyset$, such that the procedure starts with no identified changepoints. This corresponds with a large optimization penalty in PELT. Assume that we have already found $T_{k}$ to be statistically significant. We then want to check the statistical significance of the next proposed set of changepoints, $T_{k+1}$, which corresponds with a lower optimization penalty in PELT, given those changepoints in $T_{k}$. $T_{k}$ will usually, but not necessarily, be a subset of $T_{k+1}$. Figure 2A, B depicts a scenario in which we have detected changepoints $T_{k}=\{305,\;600\}$ and are evaluating $T_{k+1}=\{49,\;60,\;305,\;600\}$ as providing a significant improvement.

To assess whether $T_{k+1}$ offers a significant improvement in goodness of fit, we must both choose a goodness-of-fit measure and assess its null distribution. For goodness of fit, we use the log-likelihood of normally distributed data. More specifically, between any 2 changepoints, or between a changepoint and the start or end of the series, the observations form a “segment.” We assume that all observations are independent and normally distributed, but that those within the same segment are also identically distributed. This assumption largely follows the implementation of PELT in R's “changepoint” package.^16^

We next assess the null hypothesis that $T_{k}$ represents all of the true mean-shift changepoints in the time series using a process that may be viewed as either a Monte Carlo procedure or a parametric bootstrap. We purposefully do this in a manner that does not rely on asymptotic results, since mHealth time series can contain very small segments (see Figure 1A). We first generate a time series under the null by randomly drawing from normal distributions with the same means and standard deviations as the corresponding segments created by $T_{k}$ in the observed data. For example, Figure 2A shows the simulated data split into 3 segments by 2 changepoints at indices 305 and 600. Figure 2C illustrates a corresponding random sampling from the normal distributions whose means and standard deviations match those estimated for each of these 3 segments. We record the log-likelihood for this null time series using the $T_{k}$ changepoints. We then impose the changepoints in $T_{k+1}$ onto this null time series and calculate the corresponding log-likelihood, as depicted in Figure 2D. Finally, we record the change in the log-likelihood under the null, comparing $T_{k+1}$ with $T_{k}$.

We repeat this process N times in order to calculate an empirical *P*-value for the observed change in the log-likelihood. If the observed change is statistically significant at the chosen level, α, then we reject the null that $T_{k}$ represents all the true mean-shift changepoints for the time series, and instead select $T_{k+1}$ as the current set of significant changepoints. Figure 1B shows how the procedure continues, comparing $T_{k+1}$ to $T_{k+2}$ and so forth, until we obtain a statistically insignificant result. This hypothesis testing process is a “fixed-sequence” procedure and controls the family-wise error rate (ie, the probability of making at least one Type 1 Error across tests) at the chosen significance level, α.^17^

### ASCEPT stage 2: Trimming changepoints within linear or seasonal trends

Stage 1 identifies changepoints that include both technological changepoints, such as those associated with manufacturer updates, and changepoints from behaviorally driven patterns. In order to distinguish the former from the latter we note that, while software updates likely induce sudden mean-shifts in population-level mHealth data, behaviorally driven changes are more likely to induce linear or seasonal trends (see, eg, Figure 1A). For instance, individuals may walk more at the end of an exercise program compared to at the start (linear trend) or walk more during summer than winter (seasonal trend). These trends technically contain a mean-shift at each point, but it is unnecessary to retain these changepoints since, in practice, investigators are generally interested in identifying the start and end of these trends, and then analyzing or adjusting them in their entirety. Therefore, ASCEPT aims to exclude changepoints within trends; for convenience, we refer to these as “nuisance changepoints.” In contrast, ASCEPT retains “relevant changepoints” that correspond with a sudden mean-shift, or that are at the start or end of a linear or seasonal trend (Figure 1C). We refer to ASCEPT’s process of removing nuisance changepoints as “trimming.” Although it is the same principle as “pruning” used by methods such as CBS,^7^ we avoid the term “prune” because PELT also uses “prune” to describe part of its optimization process.^5^

**Figure 3.**
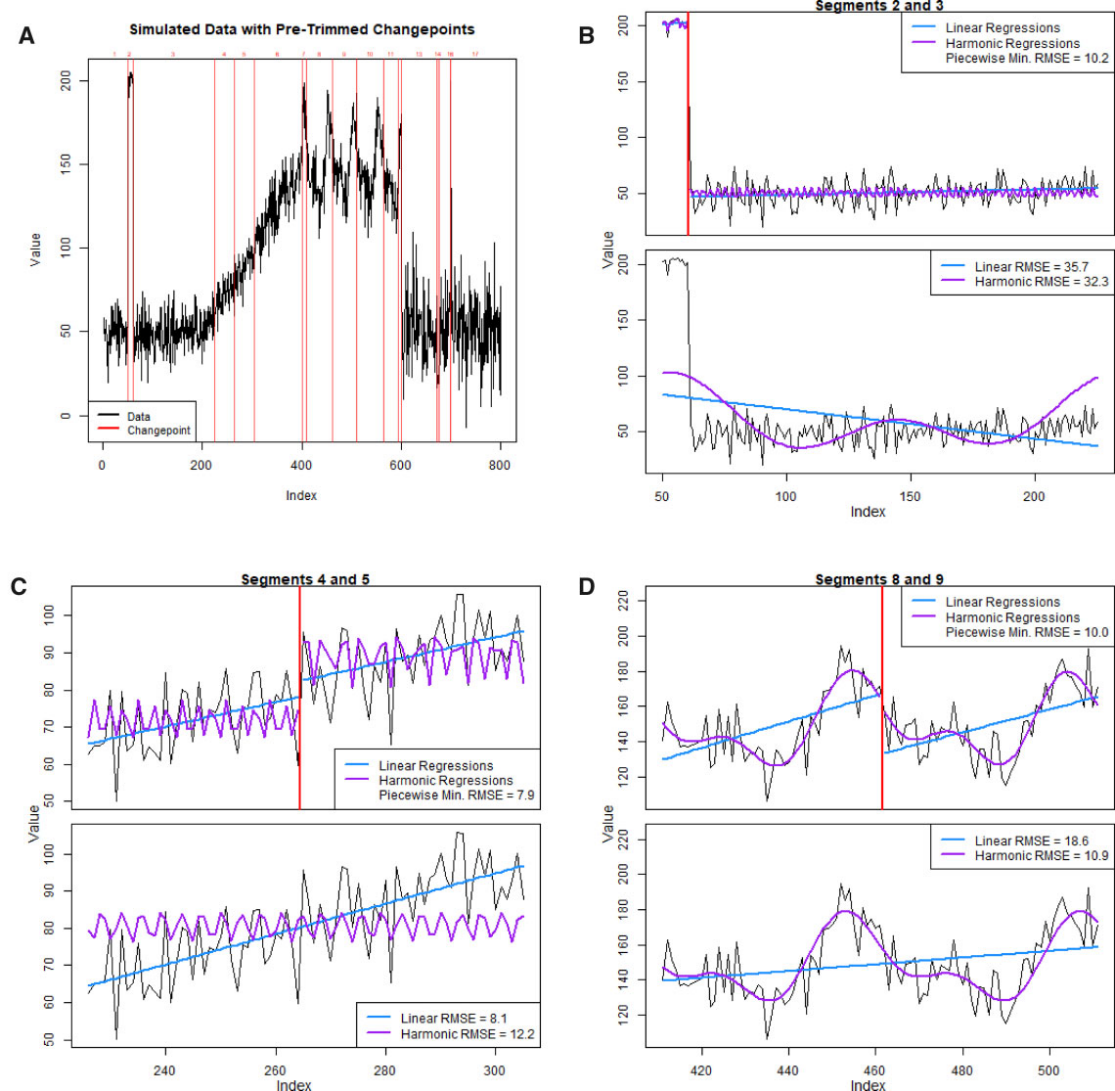
The trimming process in ASCEPT. (A) The simulated data with an initial set of changepoints. For illustrative purposes, only a subset of the changepoints found after running the first stage of ASCEPT is shown. (B) Assessing the changepoint between segments 2 and 3, a relevant changepoint. The cross-segment fits are more than 3 times worse than the best piecewise fit. (C) Assessing the changepoint between segments 4 and 5, a nuisance changepoint due to a linear trend. The cross-segment linear fit is only about 3% worse than the best piecewise fit. (D) Assessing the changepoint between segments 8 and 9, a nuisance changepoint due to seasonality. The cross-segment harmonic fit is only about 9% worse than the best piecewise fit.

We illustrate ASCEPT’s trimming process in Figure 3. Figure 3A shows a set of changepoints identified by stage 1. For every changepoint, we perform 2 types of model fits. We first fit piecewise linear and harmonic regressions on each of the 2 segments located to either side of the changepoint. We then fit linear and harmonic regressions across the 2 segments, ignoring the changepoint. To fit the linear models, which should capture linear trends, we regress the values in a segment against their indices. For harmonic regressions, which should capture seasonal trends, we first estimate a segment's period using the frequency associated with the peak of the periodogram and then fit the harmonic regression using a linear model based on this estimate. For each of the piecewise and cross-segment fits, we calculate the root mean square error (RMSE). For relevant changepoints, the piecewise fits should greatly outperform the cross-segment fits. However, for nuisance changepoints that are part of an ongoing linear or seasonal trend, the best cross-segment and piecewise fits should perform similarly.

To illustrate this, Figure 3B shows a sudden mean-shift at index 60. Here, the best piecewise fit outperforms the best cross-segment fit by nearly a factor of 3, suggesting that this is a relevant changepoint. In contrast, Figure 3C shows a nuisance changepoint that is within a linear trend. In this case, a linear regression across both segments performs only marginally worse than the best piecewise fit to the segments. Similarly, Figure 3D shows a changepoint within a seasonal pattern. In that example, the cross-segment harmonic regression performs only marginally worse than the best piecewise fit.

ASCEPT preforms this process of fitting piecewise and cross-segment models for every changepoint identified in stage 1. For each changepoint, we record the ratio of the RMSE for the best cross-segment fit to the RMSE for the best piecewise fit. The changepoint that corresponds to the smallest ratio is then removed if it falls below a chosen “trimming threshold.” This process repeats for the remaining changepoints until no ratio falls below the threshold, as depicted in Figure 1B.

### Segment correction

ASCEPT’s main purpose is to select changepoints, rather than to correct for them. However, it is important to illustrate the impact of the selection process when adjusting data downstream. Therefore, we performed a basic correction procedure. We used changepoints identified by ASCEPT and CBS to fit constant, linear, and harmonic regressions to the corresponding segments. We declared a linear or harmonic regression to be the best fit to a segment if the ratio of the constant fit's RMSE to the best corresponding linear or harmonic regression's RMSE was greater than a given “fitting threshold.” In these cases, we detrended or deseasonalized those segments. We then shifted and scaled all segments to match the location and scale of a chosen reference segment. The location was defined as the mean of the reference segment before any correction was performed and the scale was defined as the residual standard error for the best-fitting model on that segment.

### Parameters

For all main text analyses, we ran stage 1 of ASCEPT using a significance level of α=.01 and N=10 000 Monte Carlo simulations. We ran stage 2 using a trimming threshold of 1.2, such that changepoints whose best cross-segment fit had an RMSE within 20% of the best piecewise fit were subject to removal. Supplementary Figure S2 shows results from a sensitivity analysis in which we ran ASCEPT on the simulated data using various trimming threshold values.

We ran CBS, as implemented in R’s “DNAcopy” package,^18^ using a significance level of α=.01 and 10 000 permutations. We set CBS’s pruning threshold to .5; this yielded comparable results to ASCEPT’s 1.2 trimming threshold in terms of the number of changepoints identified per time series.

For segment correction, we used a fitting threshold of 1.75. We shifted and scaled with respect to the seasonal segment, as captured by either ASCEPT or CBS, as the reference.

### R package

ASCEPT is implemented in “changepointSelect,” an R package hosted on GitHub at https://github.com/matthewquinn1/changepointSelect.

## RESULTS

### ASCEPT on the simulated data

**Figure 4.**
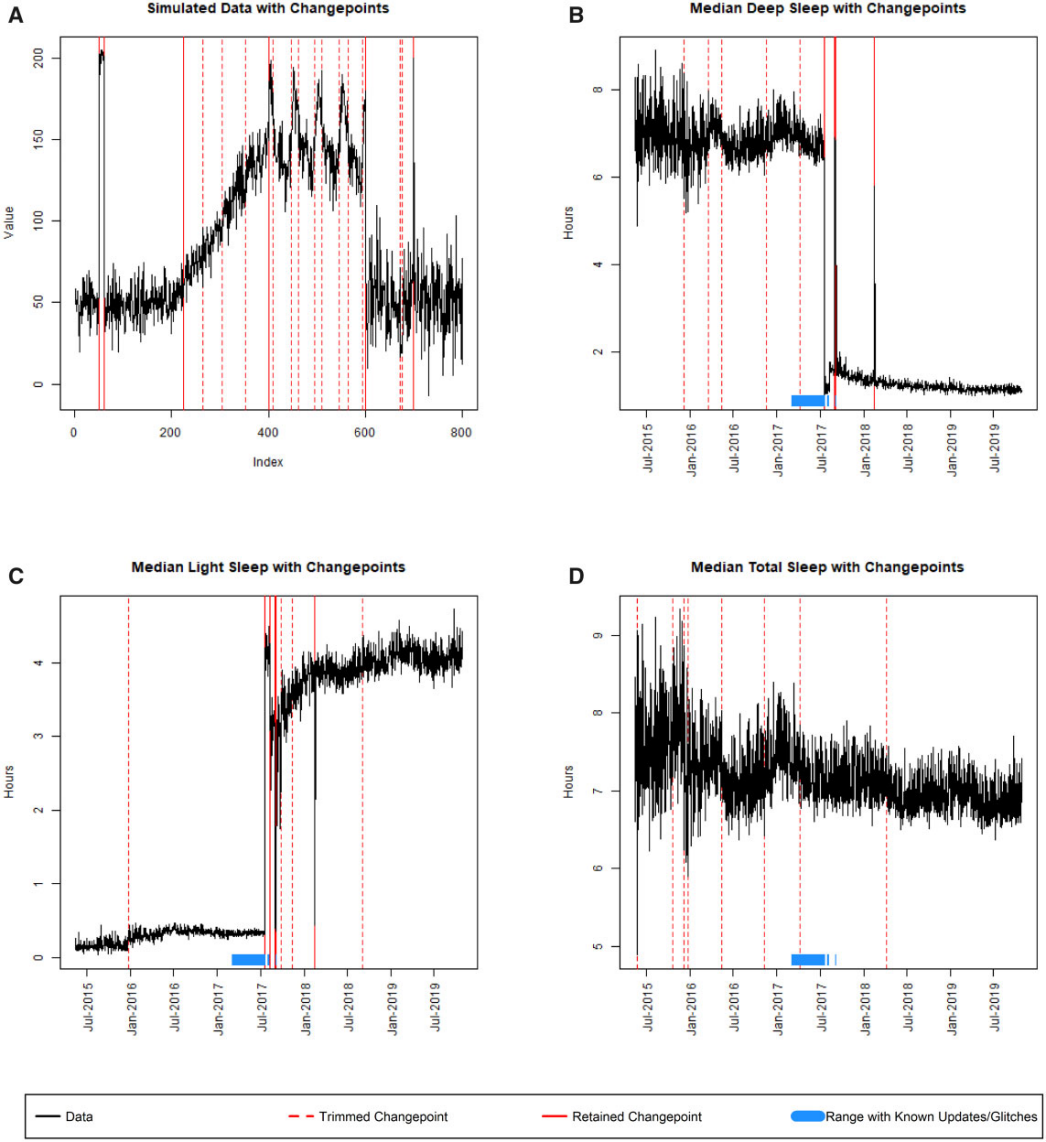
Overall results from applying ASCEPT to (A) the simulated data, as well as mHealth data from the Precisions VISSTA study measuring (B) median deep sleep, (C) median light sleep, and (D) median total sleep.

When we first applied ASCEPT to simulated data, we found that stage 1 detected 7 relevant changepoints at indices 49, 60, 225, 400, 600, 699, and 700, as well as many nuisance changepoints that were subsequently trimmed in stage 2 (Figure 4A). Comparing with known features of the simulated data (see Materials and Methods section), we observe that of these 7 detected relevant changepoints, 5 directly correspond to mean-shifts in the simulated data (at indices 49, 60, 600, 699, and 700), while the other 2 segment off the linear trend (which extended from indices 201 to 400 in the simulated data) and the seasonal trend (which extended from indices 401 to 600 in the simulated data). Thus, ASCEPT does an excellent job of identifying known changepoints in the simulated data, capturing 6 perfectly (at indices 49, 60, 400, 600, 699, and 700) and closely approximating the seventh (identified at index 225 instead of 200).

To provide an indication of ASCEPT’s runtime on the simulated data, we note that stage 1 took 14.05 seconds in serial on an 11th Gen Intel Core i7-1165G7 processor with a 2.80GHz clock speed. This decreased to 10.50 seconds when using 4 cores. Stage 2 took 0.85 seconds in serial.

### ASCEPT on the Precision VISSTA mHealth data

Next, we applied ASCEPT to mHealth data from the Precision VISSTA study. Figure 4B, C shows the results for deep and light sleep. We observed similar results for these variables, which was expected because both contribute to total sleep. For deep sleep, ASCEPT identified changepoints on July 19, 2017, September 1, 2017, September 6, 2017, February 14, 2018, and February 15, 2018. For light sleep, ASCEPT identified changepoints on July 19, 2017, August 9, 2017, August 31, 2017, September 6, 2017, February 14, 2018, and February 15, 2018. Based on this analysis, we hypothesize that Fitbit changed how it calculated sleep stage information immediately after these dates, impacting the relationship between deep and light sleep.

We further assessed these changepoints by cross-referencing with online information and found that some of the identified changepoints corresponded to known firmware updates and glitches. Alta HR received firmware update 26.62.6 between August 1, 2017 and August 10, 2017,^19^ corresponding to the August 9, 2017 changepoint for light sleep. Likewise, Fitbit modified its calculation of sleep by introducing “Sleep Stages,” starting on March 6, 2017.^20^ Users reported glitches with Sleep Stages from within a week of the release through July 24, 2017 for Alta HR, Blaze, and Charge 2 devices,^21^ encompassing the changepoint on July 19, 2017. Users again reported glitches for Blaze devices between September 3, 2017 and September 7, 2017,^22^ corresponding to the September 6, 2017 changepoint.

Cross-referencing with online information primarily allows us to investigate changepoints associated with major software/app updates announced by Fitbit, such as the introduction of the REM sleep variable in March 2017, or those that significantly impacted user experience. However, there are several changepoints that ASCEPT identified for which we did not find corresponding online information. In these cases, it is possible that Fitbit implemented minor algorithmic updates that the company felt did not warrant a public announcement and that did not cause any obvious “glitches” to individual users. We believe identifying these changepoints highlights a strength of ASCEPT. Namely, the timing and significance of mHealth algorithmic and/or device updates are not always documented and thus may be unknown to the public. At the same time, identifying and appropriately accounting for these changepoints may be important for downstream analysis.

Next, we used the daily median total sleep as a negative control (Figure 4D). While there were some large fluctuations in median total sleep during 2015, this variance was likely because relatively few individuals (as few as 7; see Supplementary Figure S1) contributed data on any given day. Accordingly, ASCEPT did not identify any changepoints in this time series after trimming. We do not suspect that Fitbit changed the calculation of total sleep during the study.

### Comparison between ASCEPT and CBS

**Figure 5.**
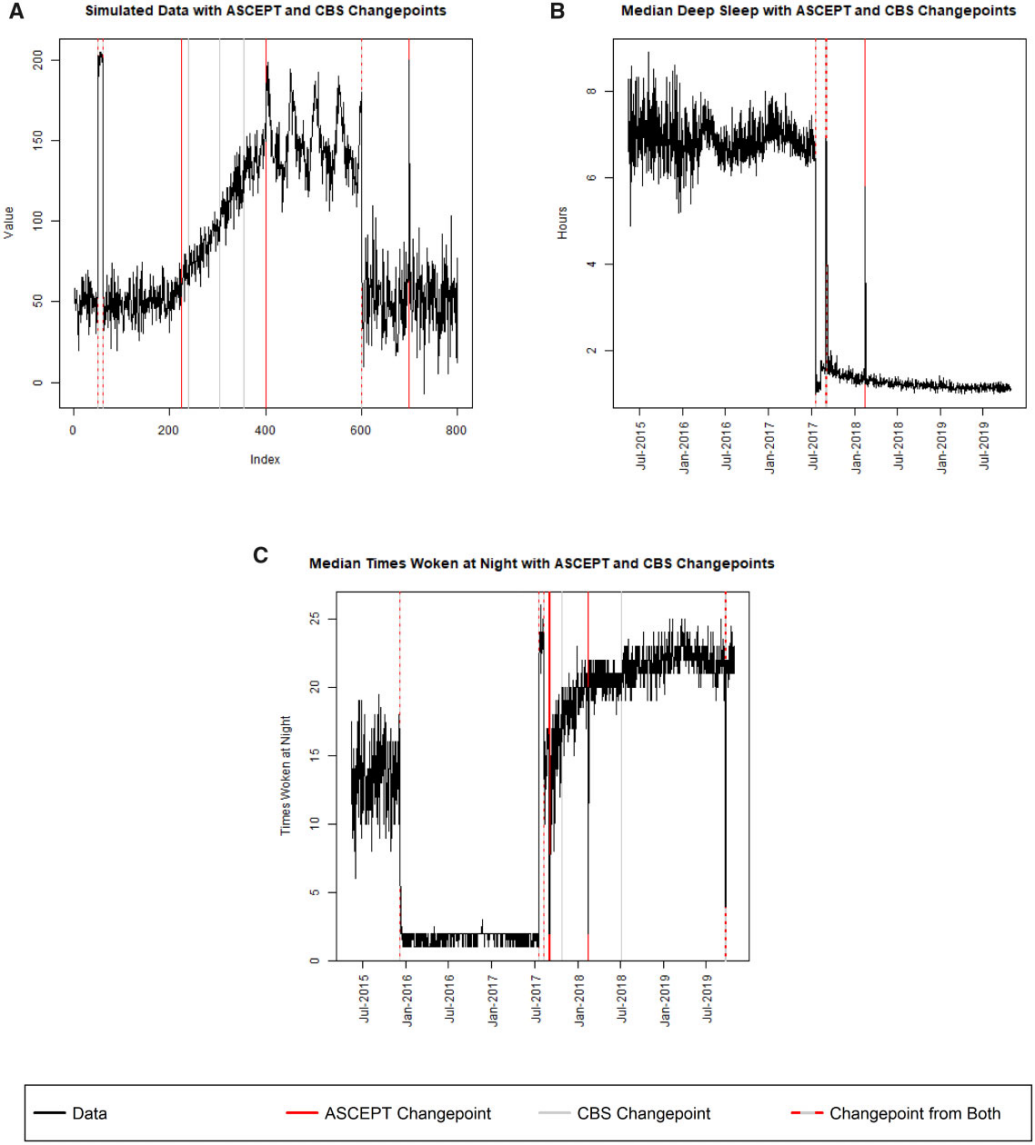
Comparison of ASCEPT with CBS when applied to (A) the simulated data, as well as mHealth data from the Precisions VISSTA study measuring (B) median deep sleep and (C) median times woken during the night.

ASCEPT shares some principles with CBS^7^ (see Supplementary Material). Therefore, we compared the changepoints identified by CBS to those identified by ASCEPT. For the simulated data (Figure 5A), we found that CBS failed to capture the single-point segment at index 700, while ASCEPT successfully did. ASCEPT also successfully segmented off the linear and seasonal trends while CBS split the linear trend into 4 segments.

We also compared ASCEPT and CBS on mHealth data from the Precision VISSTA study. For most variables, the 2 procedures yielded similar changepoints, although there were some important differences. For example, CBS failed to detect changepoints for the single-day shift in a deep sleep on February 15, 2018, while ASCEPT did (Figure 5B). The 2 procedures also greatly differed when applied to the times woken variable (Figure 5C). In particular, CBS failed to capture multiple changepoints from late 2017 to early 2018 and did not trim 2 nuisance changepoints that appeared to be within linear or seasonal trends. In contrast, ASCEPT successfully captured the major relevant changepoints and trimmed nuisance changepoints. We provide comparisons of ASCEPT and CBS for the remaining mHealth variables in Supplementary Figures S3 and S4. Visual inspection of these results demonstrates that ASCEPT generally outperformed CBS on real-world data. In some cases, ASCEPT identified several apparent changepoints that were missed by CBS (eg, for the light sleep and time awake variables), while in other cases CBS identified changepoints that did not seem well supported (eg, for the total sleep, time awake, and 4 analyzed activity variables).

**Figure 6.**
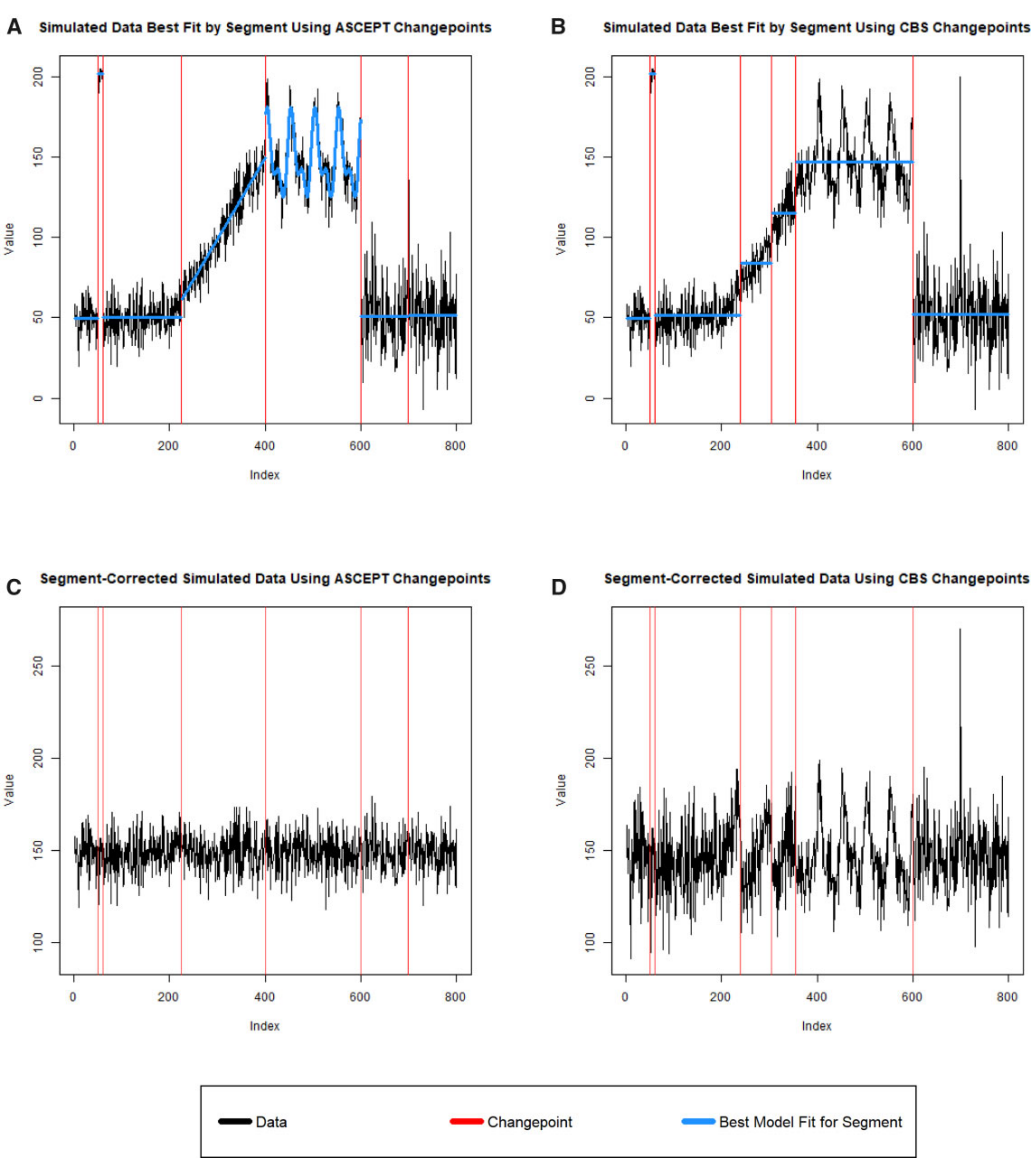
Illustration of applying a simple correction process to simulated data after identifying changepoints using either ASCEPT or CBS. (A) The best model fits using ASCEPT changepoints. (B) The best model fits using CBS changepoints. (C) The corrected series using ASCEPT changepoints. (D) The corrected series using CBS changepoints.

While ASCEPT's primary purpose is to select changepoints, we also performed a simple correction to demonstrate the importance of accurately identifying changepoints. In particular, we found the best fit model for each segment (Figure 6A, B) and then adjusted the data to match the location and scale of the segment containing the seasonal pattern, which was accurately identified by ASCEPT as indices 401–600 inclusive and identified by CBS as indices 356–600 inclusive. If the changepoints were accurately identified, then we expected the transformed time series to look like normally distributed noise without any mean-shifts. We found this to be true for the ASCEPT segment-corrected time series (Figure 6C). In contrast, the CBS segment-corrected time series (Figure 6D) still contained trends, seasonality, and other mean-shifts due to the less accurate identification of changepoints. Supplementary Figures S5 and S6 show results when using fitting thresholds other than 1.75.

## DISCUSSION AND CONCLUSIONS

We have presented an approach, ASCEPT, for identifying changepoints in mHealth data. ASCEPT builds upon the current state-of-the-art method, PELT, by incorporating the principles of statistical significance and trimming. ASCEPT adopts progressively larger sets of changepoints until the newly proposed set does not provide a statistically significant improvement in goodness of fit. ASCEPT then trims changepoints within linear and seasonal trends. In mHealth data, these trends are often attributable to human behavior or health, rather than technological issues. As a result, an investigator handling data downstream should be interested in studying these trends as a whole, rather than arbitrarily splitting them up and correcting them in the same manner as for technological changepoints. The final result from ASCEPT is a set of estimated changepoints that can be used to adjust mHealth data prior to additional downstream analyses, for example, estimating statistical associations between mHealth variables and other independently collected data, such as medical information (eg, disease diagnoses and treatment), phenotypic variables (eg, weight and blood pressure), or omics measurements.

ASCEPT offers many advantages over comparable methods. For example, using PELT to detect multiple changepoints requires specifying an optimization penalty while ASCEPT allows an investigator to specify a significance level, a more intuitive statistical parameter. Additionally, ASCEPT is specifically designed for mHealth data, which is not true of comparable methods like CBS. For instance, CBS uses a permutation test to obtain *P*-values for changepoints,^7^ but this approach has difficulty capturing segments containing only one observation, a feature we observed in the mHealth data from the Precision VISSTA study (Figure 1A). In contrast, ASCEPT's Monte Carlo procedure can capture single-point segments (Figure 4B). Additionally, CBS trims changepoints using a sum of squared within-segment deviations measure,^7^ while ASCEPT directly models the linear and seasonal trends in mHealth data, thereby helping to differentiate between common behaviorally driven patterns and other patterns that may be a result of technological changes, such as software or hardware updates to a wearable device.

Importantly, ASCEPT allows an investigator to identify potential technological changepoints automatically. For example, while Fitbit lists previous firmware versions online, it does not readily provide release dates or specific notes regarding each one.^23^ Instead, a researcher needs to manually read through online community forums for details.^24^ In our investigation of these online notes, we found that update rollouts and glitches often occurred over days or weeks, making it difficult to precisely determine when the data reflect these changes. Furthermore, some changes may not even be publicized, rendering a manual search useless. In contrast, ASCEPT provides an effective way to precisely identify when technologically driven changes occurred.

We note, however, that ASCEPT has some potential limitations. First, since it involves a Monte Carlo method, ASCEPT does not guarantee the same results over repeated runs; however, using a large number of simulations mitigates this issue. This first stage of ASCEPT also approximates *P*-values in a manner that may yield false positives because we do not rerun PELT on each simulated series when obtaining *P*-values. However, in most cases, these false positives should be removed by ASCEPT’s trimming procedure. Some time series may also be difficult for ASCEPT to analyze. For example, ASCEPT may have difficulty precisely demarcating segments within a time series that includes multiple linear trends with varying slopes. However, we found that ASCEPT generally performed well even for several challenging scenarios in the simulated and real mHealth data. ASCEPT is also computationally intensive, but we parallelized its implementation for improved performance. In addition, ASCEPT assumes that the observations are normally distributed, which may not always be true. However, normality is appropriate to use in many scenarios, such as when using the sample mean or median of a variable,^25^ as we did in our application of ASCEPT to mHealth data from the Precision VISSTA study. Lastly, ASCEPT requires the selection of 2 thresholds: a significance level and a trimming threshold. While these parameters are intuitive, we recommend that investigators use a sensitivity analysis to select a trimming threshold that is appropriate for their data. In our analyses, we found that the changepoints identified by ASCEPT were robust across a wide range of trimming threshold values.

While there are limitations, ASCEPT also has many strengths. For instance, while we developed ASCEPT for mHealth data and tested it on data from the Precision VISSTA study, the approach is generalizable. For example, a researcher could apply ASCEPT to select mean-shift changepoints in any univariate time series for which linear trends and seasonality induce nuisance changepoints. Additionally, instead of only applying ASCEPT to population-level data to identify technological changepoints, an investigator could apply ASCEPT to an individual’s time series data to identify behavioral shifts that are not associated with broader seasonal or linear patterns. One could also modify ASCEPT to identify and remove nuisance changepoints within other trends, such as quadratic trends, which may be more common in other types of data.^26^ ASCEPT could also be adjusted to handle changepoints associated with changes in variance, rather than only mean-shift changepoints, and its normality assumption could be adjusted to allow for other distributional assumptions. For example, one may want to consider a Poisson distribution for count-related mHealth variables (eg, steps) or consider lognormal, chi-squared, and gamma distributions for positive and skewed variables (eg, time active). Accounting for these in ASCEPT would entail changing the distribution used when calculating the likelihood of segments. However, since PELT assumes normality, one would also need to adjust PELT’s code. While the current presentation of ASCEPT uses PELT, a researcher could, in theory, also apply the same processes to other changepoint detection algorithms.

We designed ASCEPT as a formal process to select relevant changepoints among those proposed by PELT by using statistical tests and modeling trends that are commonly associated with nuisance changepoints. Identifying these types of changepoints is a critical step for effectively analyzing mHealth data, which often contains changepoints both from sudden changes in the propriety algorithms used to record measurements and from changes in human behavior. ASCEPT automates this process and only requires selecting 2 intuitive parameters. This affords a distinct advantage over using other methods or performing a manual identification of technological changepoints, which supports ASCEPT’s broad applicability to mHealth data analysis.

## FUNDING

National Institute of Biomedical Imaging and Bioengineering and the Office of the Director of the National Institutes of Health under award number R01EB025024 (to AC, KG, and MQ); and the National Heart, Lung, and Blood Institute under award number T32HL007427-39.

## AUTHOR CONTRIBUTIONS

AC and KG conceptualized the project. MQ and KG developed the method. MQ wrote the software and performed the formal analysis and investigation. KG and AC curated the data and resources for the project. MQ wrote the original draft. All authors reviewed and edited the manuscript. KG supervised the project. AC and KG acquired the funding.

## SUPPLEMENTARY MATERIAL

Supplementary material is available at *JAMIA Open* online.

## CONFLICT OF INTEREST STATEMENT

None declared.

## Supplementary Material

ooac090_Supplementary_Data

## Data Availability

ASCEPT’s code and the simulated data are available online at https://github.com/matthewquinn1/changepointSelect. Those interested in the availability of the Precision VISSTA data should contact AC.
