# Supplementary material for: Automated selection of changepoints using empirical P-values and trimming
Source: JAMIA Open. 2022 Oct 29;5(4):ooac090. doi: 10.1093/jamiaopen/ooac090 (PMC9617685; doi:10.1093/jamiaopen/ooac090)
Supplement: ooac090_Supplementary_Data [file ooac090_supplementary_data.pdf]

PREPROCESSING OF PRECISION VISSTA DATA

The Precision VISSTA study[1] was able to access mobile Health (mHealth) data collected through the Inflammatory Bowel Disease (IBD) Partners Patient-Powered Research Network (PPRN)[2] which allows for a wide range of wearable devices and apps to be connected to their patient portal platform. We used an API from Validic[3] to extract mHealth data from devices connected to the IBD PPRN; these data included 475,793 entries for activity variables (steps, distance, floors, elevation, calories, and active duration), and 254,276 entries for sleep variables (total sleep, light, deep, rem, awake, and times woken). Each entry was associated with a user and a time stamp. We first ensured a consistent definition of the sleep variables by confirming that the sum of awake, light, deep, and rem sleep times equaled total sleep (and adjusting if necessary). We then we removed a small number of anomalous entries in both the activity and sleep data (e.g. entries with steps>0 and calories=0 in the activity data and negative deep sleep values in the sleep data) as well as entries on dates with very low coverage (fewer than 10 total associated users). For each user, we note that a small fraction of the time multiple activity or sleep entries were associated with the same date. In these cases, we selected a single entry for a given user on each date based on the maximum steps (for activity variables) or total sleep value (for sleep variables), respectively, under the assumption that the maximum value was most likely to represent the culmination of all activity or sleep on that day. Finally, we merged entries from the activity and sleep data. This resulted in a dataset that included 315,330 merged activity/sleep entries across 522 unique users and 1,627 unique dates (~4.5 years) between May 15, 2015 and October 27, 2019. Across this preprocessed data the most common manufacturer was FitBit, with 405 users.

For the analysis in this paper, we focused on the subset of the data described above that was associated with 298 users that reported using either a heart rate (HR) Fitbit device introduced in 2016-2019 (Alta HR, Blaze, Charge 2, Charge 3, Inspire HR, Ionic, Versa, or Versa 2), multiple Fitbit devices, or an unknown Fitbit device. Since IBD PPRN uses a “bring your own device” (BYOD) approach to data collection, each user reports data across a different set of dates. Although we observed some missingness in these data, the median coverage across each individual’s usage period (i.e. the percentage of non-zero steps or total sleep values recorded between the first reported and final reported activity/sleep value for that user) was 93%. Finally, we also note that Fitbit didn’t officially introduce the capability to record REM sleep to their devices until March of 2017.[4] In our data some devices did appear to report REM sporadically in 2015 and early 2016. However, there were no values reported for REM sleep between May 20, 2016 and March 26, 2017. Therefore, we excluded this variable from our main analysis.

REVIEW OF OFFLINE CHANGEPOINT DETECTION

Researchers have created various methods for offline changepoint detection over the years. We briefly review the most relevant approaches here. In addition, [5] contains a more extensive review.

In our application for identifying changepoints in mHealth data, we are concerned with performing offline changepoint detection for an unknown number of changepoints that primarily

reflect mean-shifts in a time series. This is a common scenario for changepoint analysis and appears reasonable for mobile health research in particular.

In offline changepoint detection, the goal is typically to perform an optimization. In many cases, one will make a parametric assumption about the data, such as assuming normality. Additionally, all observations between two changepoints, which form a "segment", will be assumed to follow the same distribution, while those in segments separated by changepoints may follow different distributions, such as normal distributions with different means. Many detection algorithms identify changepoints by minimizing a cost function (e.g., negative log-likelihood) subject to a penalty for introducing additional changepoints to prevent overfitting. There are methods that provide approximate, or locally optimal, results as well as those that provide exact, or globally optimal, results. While approximate methods do not guarantee a globally optimal result, they typically offer lower computational complexities.

One of the most popular approximate methods is binary segmentation. Binary segmentation effectively considers splitting a time series of observations, $\mathrm{y}_1, \ldots, \mathrm{y}_\mathrm{T}$ for times $t = 1, \ldots, T$, into two subsegments by identifying a changepoint at time τ. To do this, the method first defines a cost function, $\mathcal{C}(\cdot)$, and sets $\tau = \mathrm{argmin}_{t\in\{1,\ldots T\}}[\mathcal{C}(y_1, \ldots, y_t) + \mathcal{C}(y_{t+1}, \ldots, y_T)]$. Here, the cost function may be something like the negative log-likelihood, if assuming a parametric model. If one wishes to detect multiple changepoints, then one can run this minimization again on each subsegment, one from $t = 1$ to $t = \tau$ and the other from $t = \tau + 1$ to $t = T$. This process repeats until some stopping criterion is met. The primary advantage of this approach is its relatively low computational complexity of $\mathcal{O}(n \log n)$ when considering a series of n observations.[6]

Other approximate approaches have built off of binary segmentation. These include Circular Binary Segmentation (CBS),[7] which allows for detection of two changepoints at a time, and Wild Binary Segmentation (WBS),[8] which randomly draws and checks segments. Though CBS is approximate, ASCEPT uses similar principles. For instance, CBS generates empirical p-values to iteratively assess potential changepoints, retaining those found to be significant. It then prunes or trims, the set of significant changepoints to remove those within linear trends. ASCEPT follows comparable principles but uses different implementations at each step.

There are also a number of exact methods for multiple changepoint detection. However, these generally suffer from relatively high computational complexities compared to approximate methods. For instance, the Segment Neighborhood method[9] has $\mathcal{O}(mn^2)$ complexity for a time series of length n with m changepoints. Likewise, the Optimal Partitioning algorithm has $\mathcal{O}(n^2)$ computational complexity.[10] The method that we consider to be the state-of-the-art is Pruned Exact Linear Time (PELT), a modified version of the Optimal Partitioning algorithm that is capable of running in $\mathcal{O}(n)$ time under certain assumptions.[6] Consider detecting m changepoints, $\tau_1, \ldots, \tau_m$, with $1 \leq \tau_1 < \cdots < \tau_m \leq n - 1$. We define $\tau_0 = 0, \tau_{m+1} = n$ for the purpose of segmenting all of the data. For a cost function, $\mathcal{C}(\cdot)$, PELT performs the minimization:

$$\min_{m,\tau_1,\dots,\tau_m} \sum_{i=1}^{m+1} \left[\mathcal{C}\left(y_{\tau_{i-1}+1}, \dots, y_{\tau_i}\right)\right] + \beta f(m)$$

Equation 1

where $f(m)$ is a penalty based on the number of changepoints and β is a multiplier on the penalty. PELT is often used with a penalty that is linear in the number of changepoints, $\beta f(m) = \beta m$. Under this condition, we can equivalently write Equation 1 as:

$$\min_{m,\tau_1,\dots,\tau_m} \sum_{i=1}^{m+1} \left[\mathcal{C}\left(y_{\tau_{i-1}+1}, \dots, y_{\tau_i}\right) + \beta\right]$$

Equation 2

PELT solves this optimization problem using dynamic programming in a similar manner to Optimal Partitioning,[10] but is able to obtain its considerable speed-up by pruning the space over which it searches for changepoints. Namely, consider the scenario where the cost function is defined to be the negative log-likelihood associated with a segment. Likewise consider indices t and s where $t < s < T$, letting $\mathcal{T}_t$ denote the set of possible changepoints to be detected over indices $1, \dots, t$ and likewise for $\mathcal{T}_s$. In the case where:

$$\min_{m,\mathcal{T}_t} \sum_{i=1}^{m+1} \left[\mathcal{C}\left(y_{\tau_{i-1}+1}, \dots, y_{\tau_i}\right) + \beta\right] + \mathcal{C}(y_t, \dots, y_s) \geq \min_{m,\mathcal{T}_s} \sum_{i=1}^{m+1} \left[\mathcal{C}\left(y_{\tau_{i-1}+1}, \dots, y_{\tau_i}\right) + \beta\right]$$

Equation 3

t cannot be the last optimal changepoint prior to T.[6] Under certain regularity conditions, notably that the expected number of changepoints increases linearly with n, this approach can achieve a complexity of $\mathcal{O}(n)$. In the worst case, PELT has the same computational complexity as Optimal Partitioning, $\mathcal{O}(n^2)$.

The main difficulty with using PELT is the specification of the penalty constant, β. Selecting β is often non-intuitive. To help with this, the Changepoints for a Range of PenaltieS (CROPS) algorithm offers an efficient approach for running PELT under many different values of β. In particular, CROPS identifies all of the different sets of changepoints detected as one varies β between a chosen β_{min} and β_{max}.[11] CROPS takes advantage of the fact that many different penalty constants will yield the same results under PELT. For instance, if a chosen β yields the set of changepoints $\mathcal{T}$, then increasing or decreasing β by a small amount will often not lead to PELT detecting fewer or more changepoints. Using CROPS, one needs to run PELT a maximum of $m(\beta_{min}) - m(\beta_{max}) + 2$ times where $m(\beta)$ refers to the number of changepoints detected under penalty constant β.

Running CROPS on PELT allows an investigator to explore the results from PELT under many different penalties. However, this approach still suffers from some practical challenges. For example, CROPS gives an investigator the results of many runs of PELT but does not provide any indication as to which set of changepoints is the “best” set among those runs. The investigator has to manually determine which set is the most appropriate for their data. Thus, we need an approach for selecting an optimal set among those presented by CROPS. This is especially difficult to formalize when investigating multiple time series, such as what we encountered in our analysis of mHealth data from the Precision VISSTA study. There is clearly a

need for a rigorous approach for selecting a final set of changepoints in this context. This is the primary motivation for ASCEPT.

ADDITIONAL SIMULATED DATA RESULTS FOR VARIOUS TRIMMING THRESHOLDS

In the main manuscript, we present the results of ASCEPT when using a trimming threshold of 1.2. However, it is important to note that our specific results depended on this selected threshold value. We investigated which changepoints ASCEPT retained or trimmed when varying the trimming threshold for the simulated time series data (**Supplementary Figure 2**). We found that any trimming threshold between 1.13 and 1.20 inclusive yielded the same final set of changepoints while a trimming threshold greater than 1.20 trimmed out the changepoints from Stage 1 of ASCEPT at indices 699 and 700, thereby introducing false negatives. Decreasing the trimming threshold below 1.13 resulted in ASCEPT retaining multiple changepoints initially detected within the seasonal pattern between indices 401 and 600 inclusive, thereby introducing nuisance changepoints. Overall, this analysis shows that, while results are fairly robust across multiple trimming thresholds, it is important to choose an appropriate value in order to avoid either removing or retaining too many changepoints.

ADDITIONAL PRECISION VISSTA RESULTS FOR ASCEPT AND CBS

In **Supplementary Figures 3** and **4**, we present the results for both ASCEPT and CBS on different variables from the Precision VISSTA study, excluding those in **Figure 5** of the main text. Across the different variables, we found that ASCEPT generally outperformed CBS at identifying mean-shifts in the data, especially those lasting only one day, and at trimming changepoints within linear and seasonal trends.

While ASCEPT performed well when applied to these various time series, we identified one exception when investigating the awake variable, depicted in **Supplementary Figure 3C**. Here, both ASCEPT and CBS missed four relevant changepoints. In the case of ASCEPT, reducing the trimming threshold to 1.15 resulted in the method capturing two of these changepoints. Interestingly, the behavior of this variable was nearly identical to the times woken variable, on which ASCEPT performed well (see **Figure 5C** in the main text). This indicates that small changes in a series can sometimes yield fairly different results in the final set of identified changepoints. We note that changing the trimming threshold to 1.15 also introduced several nuisance changepoints in the series of times woken, emphasizing the importance of considering multiple trimming thresholds.

ADDITIONAL RESULTS FOR SEGMENT CORRECTION

The main text’s segment correction analysis used a fitting threshold of 1.75. A linear or harmonic regression was deemed the best fit to a segment only if the ratio of the constant fit's RMSE to the best corresponding linear regression or harmonic regression's RMSE was greater

than this fitting threshold. **Supplementary Figures 5** and **6** show the results when using 1.50 and 1.25 as fitting thresholds**,** respectively.

The results did not change appreciably when performing segment correction using the ASCEPT-identified changepoints. The only difference was that under fitting thresholds of 1.50 and 1.25, the segment from indices 50 to 60 was incorrectly identified to be best fit with a harmonic regression, rather than a constant fit. This segment was therefore transformed slightly differently than it was in **Figure 6C**. Despite this change, the transformed series under ASCEPT changepoints still appeared to be normally distributed noise without any mean-shifts. **Supplementary Figures 5A**, **5C**, **6A** and **6C** show these results.

For CBS, the linear and seasonal trends were more appropriately modeled using the smaller fitting thresholds, as shown in **Supplementary Figures 5B**, **5D**, **6B**, and **6D**. In particular, along the segment corresponding to the seasonal trend, the best fit was now a harmonic regression. Since all segments are scaled to match the residual standard error of this chosen reference segment, the transformed series in **Supplementary Figures 5D** and **6D** had smaller spreads than that shown in **Figure 6D**, where the best fit for this segment was identified as a constant trend. However, there were still some issues with the segment correction due to CBS' misidentification of the relevant changepoints. There were clear residual mean-shifts, including linear trends between indices 201 and 400 and the single-point segment at index 700.

Overall, we found that, while this correction procedure was somewhat sensitive to the chosen fitting threshold, the accurately identified ASCEPT changepoints were more robust to the choice of threshold and yielded more ideal downstream results compared to the less accurate CBS changepoints.

Supplemental References

1 Chung A, Gotz D, Kappelman M, *et al. Precision VISSTA: Enabling Precision Medicine through the Development of Quantitative and Visualization Methods*. http://precisionvissta.web.unc.edu/

2 Chung AE, Sandler RS, Long MD, *et al.* Harnessing person-generated health data to accelerate patient-centered outcomes research: the Crohn's and Colitis Foundation of America PCORnet Patient Powered Research Network (CCFA Partners). *J Am Med Inform Assoc* 2016;**23**:485–90. doi:10.1093/jamia/ocv191

3 Validic. Validic API Documentation. 2016.https://docs.validic.com/v1.2016-03-01/docs

4 Kosecki D. New Fitbit Features Deliver Data Previously Only Available Through a Sleep Lab. 2017.https://blog.fitbit.com/sleep-stages-and-sleep-insights-announcement/

5 Truong C, Oudre L, Vayatis N. Selective review of offline change point detection methods. *Signal Process* 2020;**167**:107299. doi:https://doi.org/10.1016/j.sigpro.2019.107299

6 Killick R, Fearnhead P, Eckley I. Optimal Detection of Changepoints With a Linear Computational Cost. *J Am Stat Assoc* 2012;**107**:1590–8. doi:10.1080/01621459.2012.737745

7 Olshen A, Venkatraman ES, Lucito R, *et al.* Circular binary segmentation for the analysis of array-based DNA copy number data. *Biostatistics* 2004;**5**:557–72. doi:10.1093/biostatistics/kxh008

8 Fryzlewicz P. Wild binary segmentation for multiple change-point detection. *Ann Stat* 2014;**42**:2243–81. doi:10.1214/14-AOS1245

9 Auger I, Lawrence C. Algorithms for the optimal identification of segment neighborhoods. *Bull Math Biol* 1989;:39–54. doi:10.1007/BF02458835

10 Jackson B, Scargle JD, Barnes D, *et al.* An Algorithm for Optimal Partitioning of Data on an Interval. *IEEE Signal Process Lett* 2005;**12**:105–8. doi:10.1109/LSP.2001.838216

11 Haynes K, Eckley IA, Fearnhead P. Computationally Efficient Changepoint Detection for a Range of Penalties. *J Comput Graph Stat* 2017;**26**:134–43. doi:10.1080/10618600.2015.1116445

Supplementary Figure Captions

Supplementary Figure 1. (A) The daily median total sleep from the Precision VISSTA study and (B) the corresponding number of contributing observations each day.

Supplementary Figure 2. The simulated time series with ASCEPT changepoints initially detected, using a 0.01 significance level and 10,000 Monte Carlo simulations, trimmed at various thresholds. All changepoints are retained at a threshold of 1, and all are removed by a threshold of 1.5. Thresholds between 1.13 and 1.2 inclusive all yield the same results as a threshold of 1.2, as used in the main manuscript.

Supplementary Figure 3. Comparison of ASCEPT with CBS for (A) median light sleep, (B) median total sleep, and (C) median time awake at night.

Supplementary Figure 4. Comparison of ASCEPT with CBS for (A) median time active, (B) median calories burned, (C) median distance walked, and (D) median steps.

Supplementary Figure 5. The results of performing segment correcting using a 1.50 fitting threshold. (A) The best model fits using ASCEPT changepoints. (B) The best model fits using CBS changepoints. (C) The corrected series using ASCEPT changepoints. (D) The corrected series using CBS changepoints.

Supplementary Figure 6. The results of performing segment correction using a 1.25 fitting threshold. (A) The best model fits using ASCEPT changepoints. (B) The best model fits using CBS changepoints. (C) The corrected series using ASCEPT changepoints. (D) The corrected series using CBS changepoints.

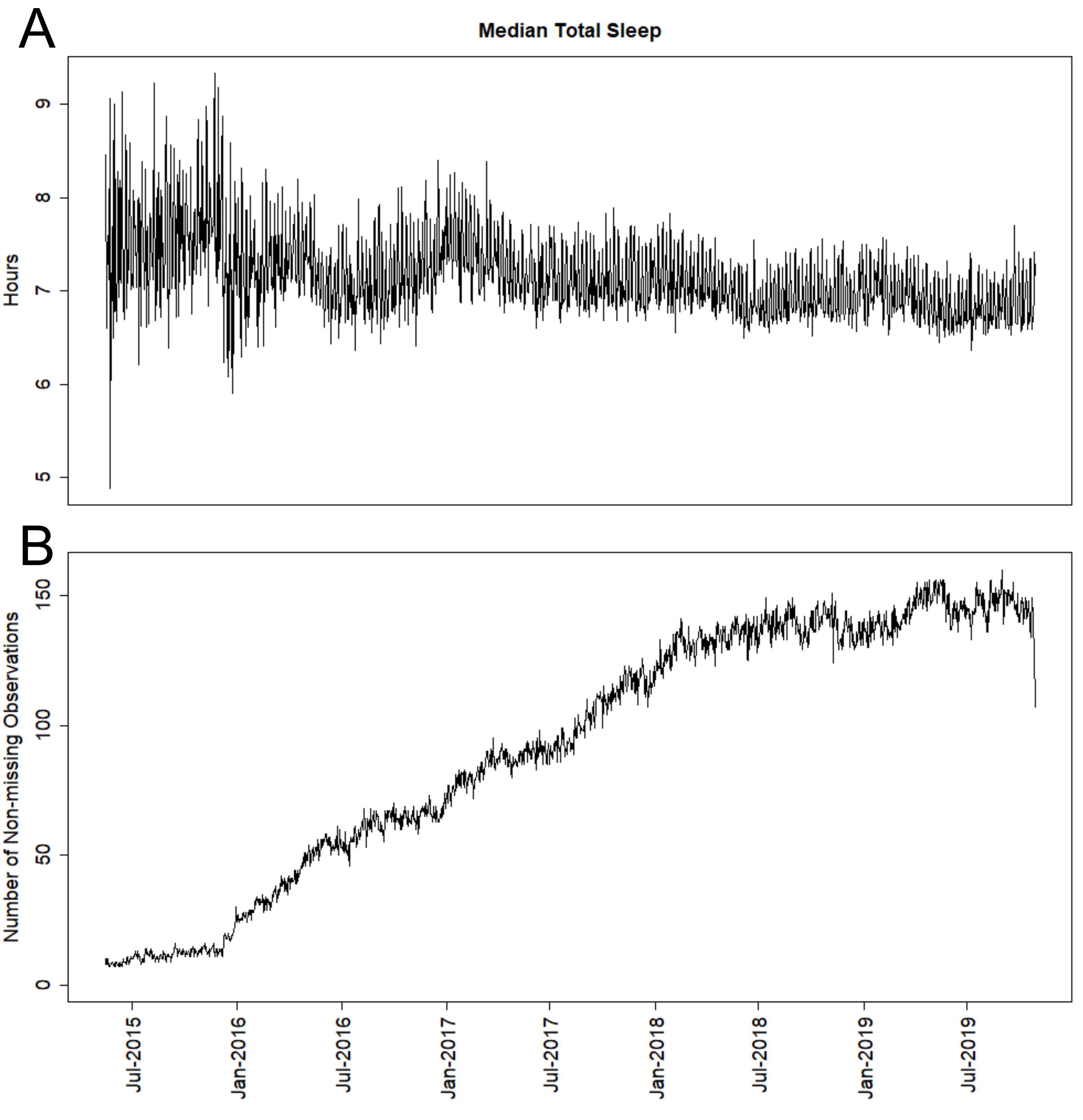

Supplementary Figure 1. (A) The daily median total sleep from the Precision VISSTA study and (B) the corresponding number of contributing observations each day.

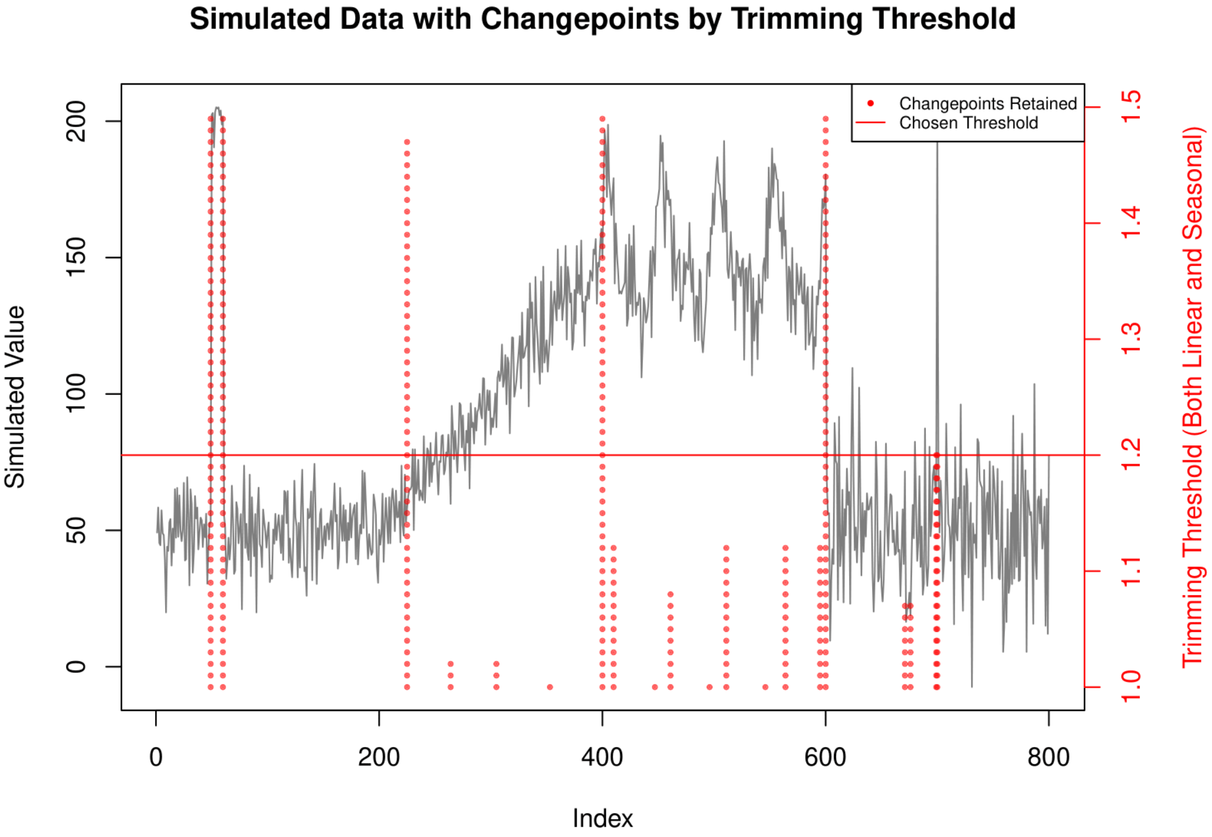

Supplementary Figure 2. The simulated time series with ASCEPT changepoints initially detected, using a 0.01 significance level and 10,000 Monte Carlo simulations, trimmed at various thresholds. All changepoints are retained at a threshold of 1, and all are removed by a threshold of 1.5. Thresholds between 1.13 and 1.2 inclusive all yield the same results as a threshold of 1.2, as used in the main manuscript.

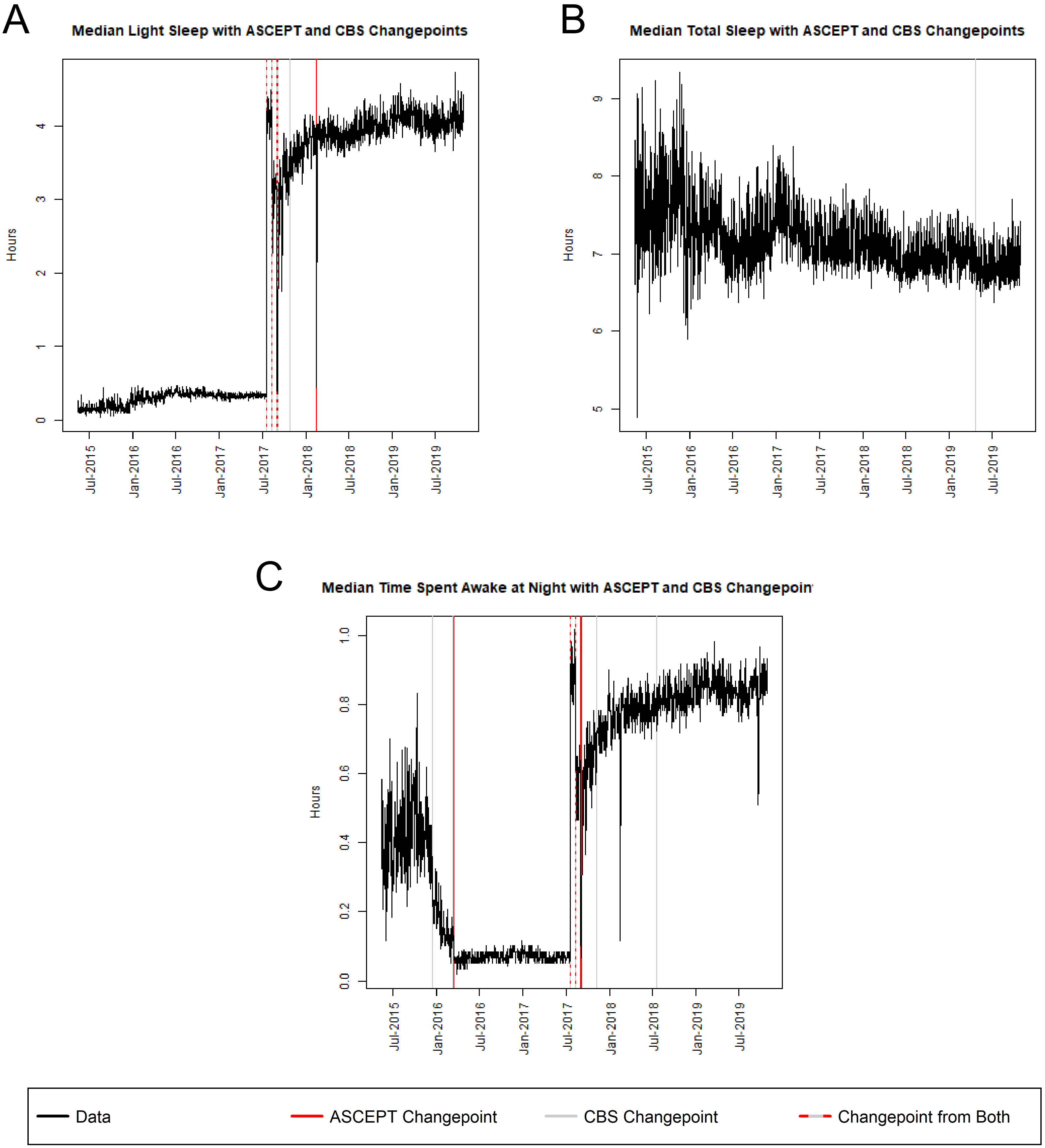

Supplementary Figure 3. Comparison of ASCEPT with CBS for (A) median light sleep, (B) median total sleep, and (C) median time spent awake at night.

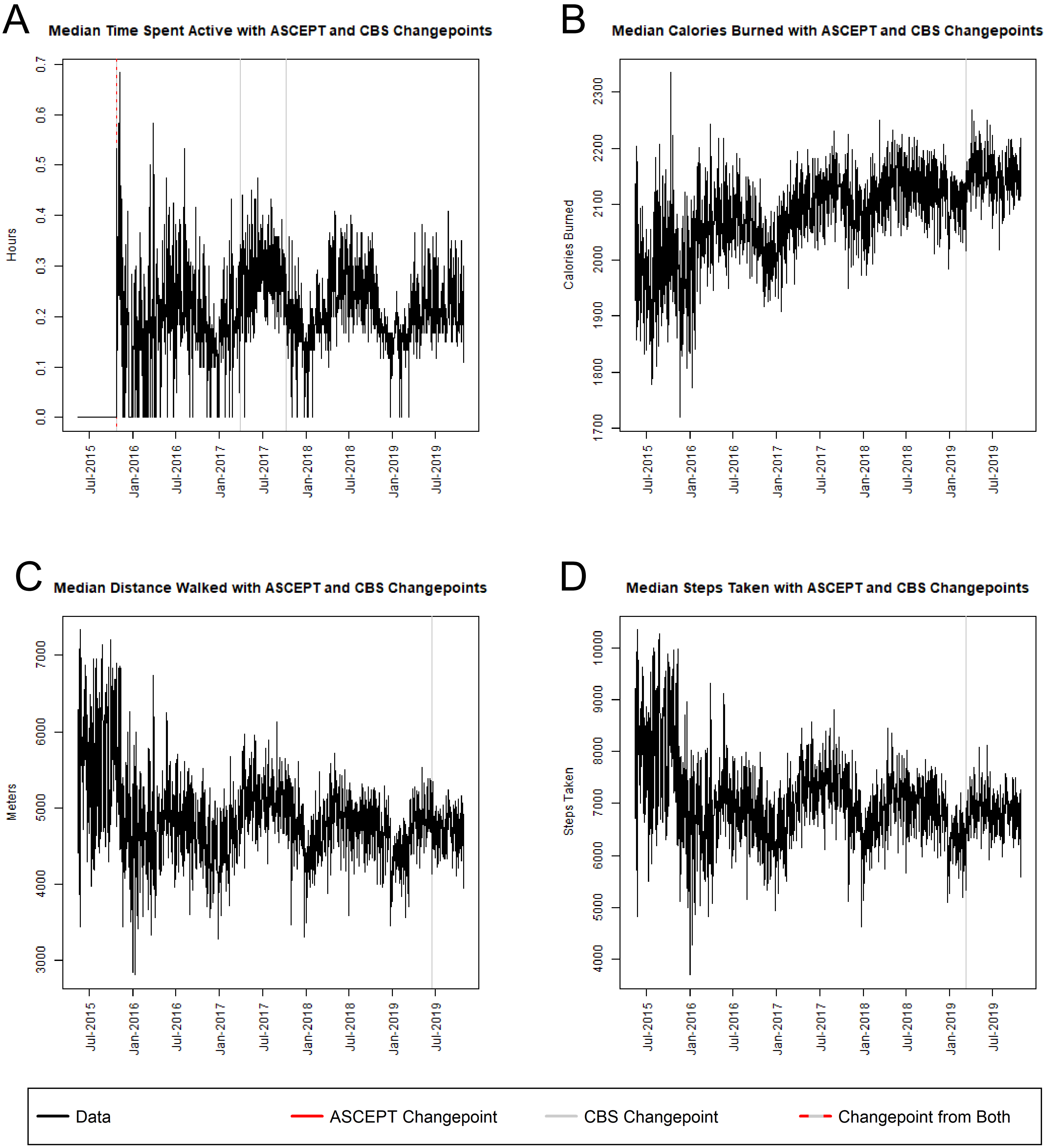

Supplementary Figure 4. Comparison of ASCEPT with CBS for (A) median time spent active, (B) median calories burned, (C) median distance walked, and (D) median steps.

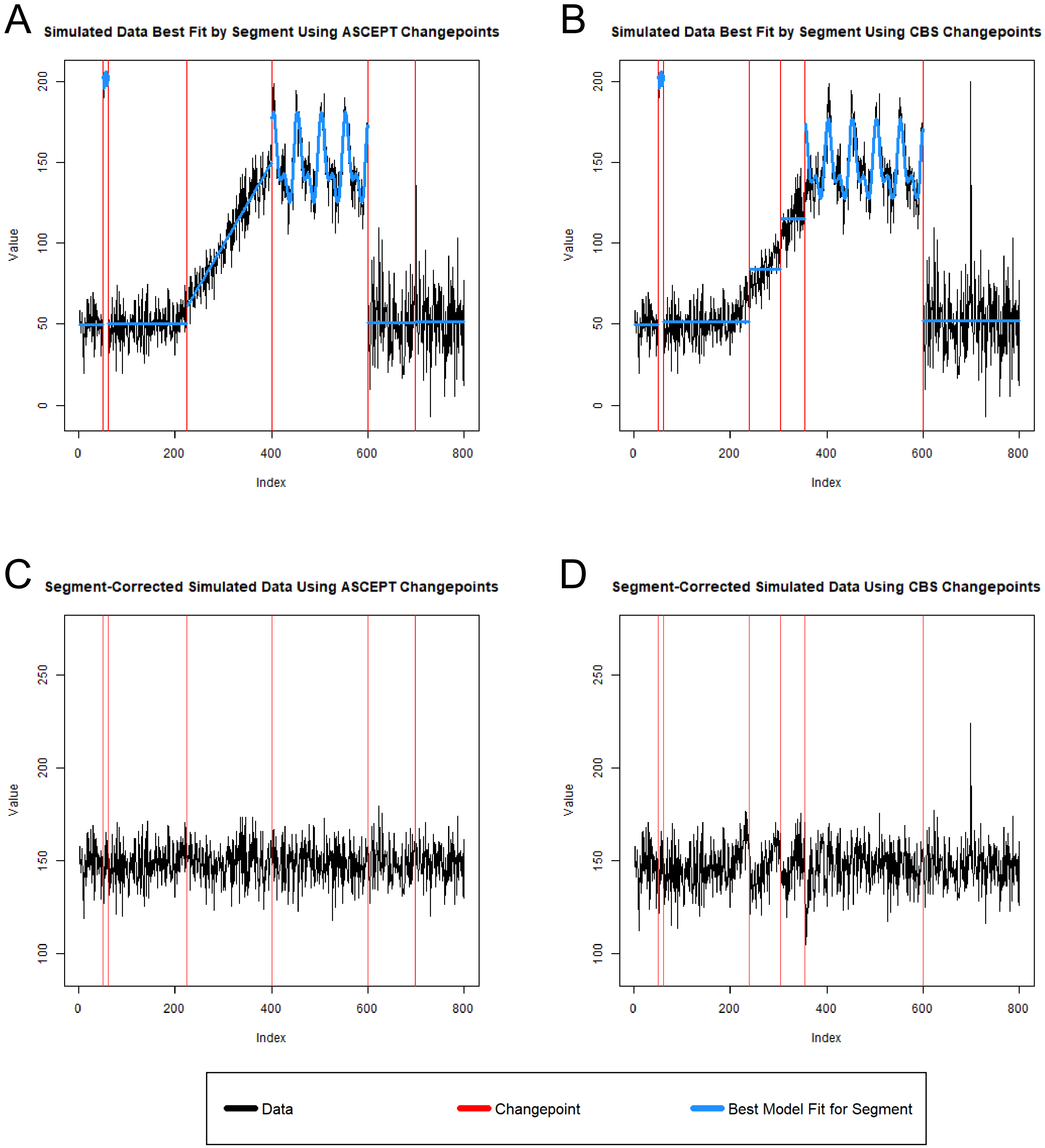

Supplementary Figure 5. The results of performing segment correcting using a 1.50 fitting threshold. (A) The best model fits using ASCEPT changepoints. (B) The best model fits using CBS changepoints. (C) The corrected series using ASCEPT changepoints. (D) The corrected series using CBS changepoints.

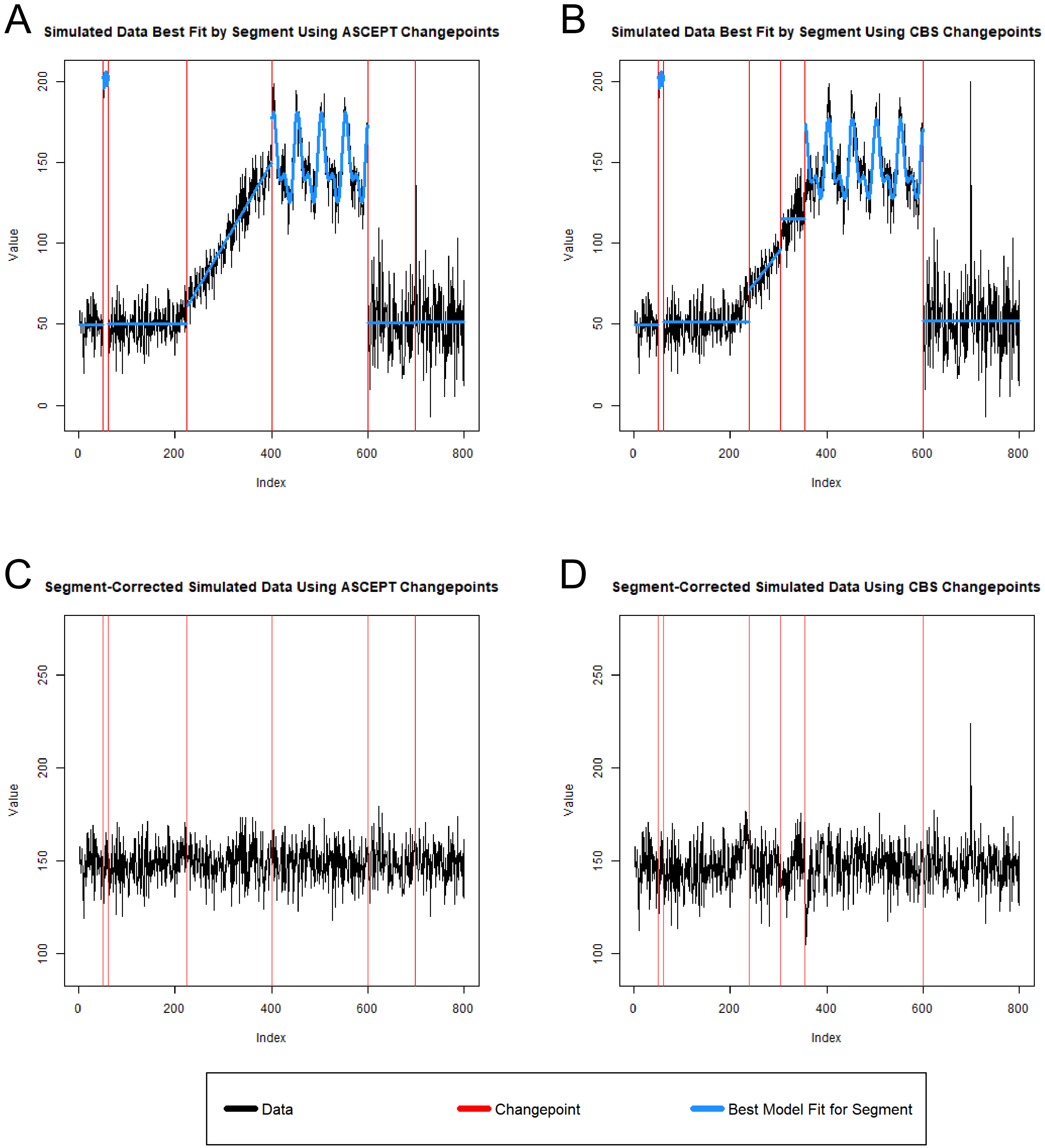

Supplementary Figure 6. The results of performing segment correction using a 1.25 fitting threshold. (A) The best model fits using ASCEPT changepoints. (B) The best model fits using CBS changepoints. (C) The corrected series using ASCEPT changepoints. (D) The corrected series using CBS changepoints.
